# The 33.1 kDa Excretory/secretory Protein Produced by *Toxocara canis* Larvae Serves as a Potential Common Biomarker for Serodiagnosis of Toxocariasis in Paratenic Animals and Human

**Published:** 2017

**Authors:** Huu-Hung NGUYEN, Doan-Trung VO, Thi-Tuyet-Trinh THAI, Thi-Thanh-Thao LE, Thanh-Dong LE, Nghia-Son HOANG

**Affiliations:** 1. Institute of Tropical Biology, Vietnam Academy of Science and Technology (VAST), Ho Chi Minh, Vietnam; 2. Nguyen Tat Thanh University, Ho Chi Minh, Vietnam; 3. Institute of Malariology, Parasitology and Entomology, Ho Chi Minh, Vietnam

**Keywords:** *Toxocara*, Excreted/secreted proteins, IgG response, Paratenic hosts

## Abstract

**Background:**

Toxocariasis is a prevalent zoonosis disease caused by the closely related nematode species *Toxocara canis* and *Toxocara cati* which parasitise Canidae and Felidae respectively. In paratenic hosts, larvae of these worms cause multiple organ damage. However, how these paratenic hosts response to these worms and whether any common biomarker can be applied for diagnosis are still unclear.

**Methods:**

Excreted/secreted (E/S) antigens were prepared by culture of *T. canis* larvae in vitro. Using a western blot (WB) assay the humoral IgG responses, induced by *Toxocara* spp. larvae to the worm’s E/S antigens in different infected hosts including mice, rabbits and human, were examined.

**Results:**

In a mouse model of toxocariasis, intraperitoneal injection of *T. canis* larvae induces inflammatory leukocyte accumulation in the liver and the lungs but not in the brain, although a remarkable number of larvae were detected in this organ. Mice and rabbits responded differently to *Toxocara* spp. resulting in distinct heterogenous WB band patterns. Mice and rabbits both responded to a 33.1 kDa E/S constituent that turned out to be the most sensitive protein for serodiagnosis. Sera from human toxocariasis patients showed heterogenous WB band patterns similar to those observed in rabbits and all responded to the 33.1 kDa band.

**Conclusion:**

33.1 kDa E/S protein can be considered as a critical common biomarker for toxocariasis immuno-diagnosis in both paratenic animals and human and its specificity requires further investigation.

## Introduction

Toxocariasis is a disease caused by infection with the nematodes *Toxocara canis* and *Toxocara cati* whose definitive hosts belong to the Canidae and Felidae respectively. In these hosts, the nematodes complete their development. In human and others animals, the development cycle of the nematodes is limited to the second larval stage which remains in the tissues for a long period of time (1). In humans, depending on where the larvae migrate, toxocariasis can present as visceral larva migrans (VLM), ocular larva migrans (OLM), and neural larva migrans (NLM) or as covert or common toxocariasis (CT). Toxocariasis is a neglected disease and the number of afflicted people may be currently seriously underestimated. In Vietnam, there is a very high toxocariasis infection rate of approximately 30% in patients who visited hospitals (2). In Mexico, sero-prevalence was 17.59% in children (12–16 yr old age) (3). In the USA 13.9% of people > 6 yr old age were infected (4). In Brazil, 38.8% of schoolchildren were infected (5). In Iran the infection level was 15.8% (6), and in Argentina 31.6% (7).

Because of the restriction in the development cycle and the uncontrolled migration of larvae into different tissues in the paratenic hosts, symptoms of infection are nonspecific and this makes the diagnosis of toxocariasis difficult. Immunological approaches, therefore, have been developed to detect specific antibodies binding to toxocariasis antigens (8). Both the excreted/secreted (E/S) antigens and the crude antigens derived from *T. canis* larvae have been intensively studied (9–11). Using a WB assay, the proteins of 24–35 kDa in the E/S antigens of *T. canis* have 100% reactivity and 98.6% specificity, whereas, higher MW E/S proteins show a number of cross-reactions to other helminthic diseases (12). However, detection of specific antibodies still cannot discriminate current and past infections. Therefore, detection of *Toxocara* (E/S) antigens in the circulation has been suggested as a mean to detect current toxocariasis (13, 14). Though this approach has high specificity (100%), its reactivity is low (31%) (14). This antigen detection assay has a drawback in the period of infection since free circulation antigens might be rapidly removed by neutralizing antibodies generated after infection. This was the case in human fascioliasis in which the *Fasciola hepatica* E/S proteins were clearly reduced after a given time of infection (15). In addition, the number of larvae involved in the infection can decisively affect the results of this analysis because only pg levels of E/S antigens are produced per larva per day (16). In addition, discrimination between the two species of *Toxocara* remains a problem.

E/S proteins produced by larvae have been suggested to play a vital role in the pathogenesis of *Toxocara* spp. (17). High protease activities were found in the high MW constituents (≥120 kDa) that can degrade a range of functional proteins including gelatin, laminin, fibronectin, albumin and IgG suggesting that the larvae use proteolysis as a strategy to protect themselves against host molecular attack. E/S proteins induced the anti-inflammatory cytokines (IL-4, IL-5, IL-6, IL-10 and TGF-β) but not the inflammatory cytokines (TNF-α, IFN-γ, and IL-17) in re-stimulated splenocytes derived from *T. canis* infected mice and that mucins in the E/S product dominantly contribute to this immune stimulation (18). Other study showed that IL-5 mRNA expression levels increases in the brain of infected immunocompetent mice due to the migration of larvae into the organ (19).

By modulating the immune system, E/S proteins induce the production of specific IgG in infected organisms. However, the way in which the different paratenic hosts respond to these E/S proteins and a common biomarker that can be applied for serodiagnosis are largely unknown. This study therefore focuses on the analysis of humoral responses of distinct hosts including mice, rabbits and human infected with *T. canis* and/or *T. cati* to find out which E/S constituent can be a potential candidate for serodiagnosis in both paratenic animals and human.

## Materials and Methods

### Animals

Swiss outbred mice (albino) (20–25gr in weight) and rabbits (2kg in weight) were purchased from the Pasteur Institute in Ho Chi Minh City, Vietnam and then kept in our laboratory (Lab for Animal Biotechnology) during experiments. Pups and kittens (2–3 months old) were purchased in Ninh Thuan Province and Ho Chi Minh City, respectively, Vietnam.

All procedures performed in studies involving animals were in accordance with the ethical standards of the Local Science and Ethical Committee at the Institute of Tropical Biology.

### Patient sera

Toxocariasis human sera were a gift from Prof. Tran Thi Kim Dung (Parasitology Department, Medicine and Pharmacy University Ho Chi Minh City, Vietnam). Cord blood sera from volunteer women were used as negative control.

### T. canis and T. cati adult worm and egg collection

Pup and kitten feces were sampled to test for the presence of *Toxocara* spp. eggs by phase contrast microscopy. Infected animals were anesthetized with ketamine (100 mg/kg of body weight). *Toxocara* spp. adult worms found in the small intestine were collected in pre-warmed PBS buffer. The worms were then washed several times in 1% formalin-PBS buffer. For egg production, all male and female worms were cultured together in PBS supplemented with 1% human serum plus penicillin (100U/ml) and streptomycin (100ug/ml) at 37 °C in a 5% CO_2_ atmosphere for up to 7 days. The eggs were collected by centrifugation at 200-x g for 10 min and then counted.

### Preparation of T. canis and T. cati infective larvae

Worm eggs were incubated in 1% formalin– PBS for 30 d at room temperature in a sterile flask. The fertilized eggs and embryo development were followed using an inverted microscope. Hatching was induced using a modified version of a published protocol (20). Briefly, eggs were incubated in 6% sterile NaClO solution for 5min at room temperature to remove the external chitinous layer and then washed twice with sterile PBS buffer. After each wash the eggs were collected by centrifugation at 200-x g for 10min. Eggs were incubated with sterile Hank’s balance saline solution pH 2.0 (HBSS) containing 5.4mM KCl, 0.3mM Na_2_HPO_4_, 0.4mM KH_2_PO_4_, 1.3mM CaCl_2_, 0.5mM MgCl_2_.6H_2_O, 0.6mM MgSO_4_.7H_2_O, and 137mM NaCl for 30 min at room temperature. The pH was adjusted to 7.4 using NaHCO_3_ and then to pH 2.0 with 5N HCl. The treated eggs were washed with sterile HBSS pH 7.4, concentrated by centrifugation at 200-x g for 10min and incubated in serum free DMEM medium supplemented with penicillin (100U/ml) and streptomycin (100ug/ml) at 37 °C in a 5% CO_2_ atmosphere. Three days later, >95% of fertilized eggs had hatched. Larvae were concentrated by centrifugation and living larvae were selected by letting them pass through a 40um cell strainer in DMEM medium overnight. All dead and weak larvae and unfertilized eggs remained in the upper side of the cell strainer. Our improved filtration method helped to obtain only strong and fast moving larvae. These were then used to produce E/S protein.

### *T. canis* excretory/secretory protein preparation

3.7 × 10^5^ *T. canis* larvae were cultured at a density of 1000 larvae/ml in DMEM medium in the presence of penicillin (100U/ml) and streptomycin (100ug/ml) at 37 °C in a 5% CO_2_ atmosphere. Culture medium was harvested every four weeks for at least one year. 1ml of culture medium was dialyzed in PBS and the protein concentration was determined by Bradford assay. E/S proteins in the culture medium were concentrated by centrifugation with the Amicon centrifugal filter units (10 kDa cut off). A cocktail of protease inhibitors (2mM AEBSF, 0.3 μM Aprotinin, 130 μM Bestatin, 1 mM EDTA, 14 μM E-64, 1 μM Leupeptin) (Sigma, Missouri, USA) was added to E/S proteins which were then stored at −20 °C until use.

### Animal models

Mice (N =5–10) and rabbits (N=6) were infected with 1000 living larvae of *T. canis* or *T. cati* by intraperitoneal injection (ip) (21–25). The same numbers of heat-treated (dead) larvae were applied ip to other groups of mice (N=3) and rabbits (N=2). Four to twenty four weeks later, sera from these animals were collected and kept at −20 °C in the presence of 0.05% NaN_3_. Sera obtained from untreated animals (N=3) were used as negative controls.

### Histological analysis and recovery of larvae in the mouse brain after infection

Four weeks after infection in mice, organs including the liver, the lungs and the brain were harvested. Half of the brain was used to recover migratory larvae by using the Baermann funnel method. For histological analysis, the half of the brain and other tissues were embedded in paraffin. 3um thick sections were then prepared and stained with Giemsa staining solution (Sigma-Aldrich, Buchs, Switzerland). Images were obtained using an OPTIKA microscope equipped with an OPTICAM B5 camera (OPTICA SRL, Ponteranica, Italy).

### SDS-PAGE

E/S proteins (0.2μg to 2.4μg/well) were electrophoresed by SDS-PAGE under reducing condition on a 10% gel. Page Ruler broad range unstained protein ladder (Pierce Biotechnology, Illinois, USA) was used as molecular weight standards. For protein detection, silver staining was used. Protein molecular weight was estimated by using Quantity One software ver. 4.6.3 (Bio-Rad Laboratories, California, USA).

### Western blotting

After SDS-PAGE, E/S proteins were transferred to a nitrocellulose membrane (0.45um of pore size) using a semi-dry blotter system (TE70XP Semi-Dry Transfer Unit, Hoefer Inc, Massachusetts, USA). For optimization of the protein transfer, blotting times of 60min, 90min, or 120min were tested. The membrane was then blocked with 0.1% casein in TBST buffer (20mM Tris, 150mM NaCl, 0.1% Tween 20, and pH 7.5) for 1hr and washed three times in TBST buffer for 10min. The membrane was incubated for 1h with *T. canis* infected mouse or rabbit or human sera diluted 1:1000 in TBST and then washed with TBST buffer. The membrane was incubated with a 1:5000 diluted goat anti-mouse or anti-rabbit or anti-human IgG antibodies conjugated with HRP (Santa Cruz Biotechnology, Texas, USA) for 1h and then washed with TBST buffer. For detection of antigen-antibody immune complexes, the membrane was incubated in enhanced chemiluminescence reagent for 5min and then scanned on C-digit blot scanner (LI-COR Biosciences, Nebraska, USA) to obtain images which were analyzed by Image Studio Lite programme.

In some experiments, western blot membrane strips were prepared as described elsewhere (12) but with modifications. Briefly, for the SDS-PAGE, a comb with wide (57.5mm width) teeth was used and 16.4μg of E/S proteins was loaded (0.28μg/mm). After electrophoresis, proteins in the gel were transferred onto a nitrocellulose membrane that was then blocked with blocking solution, air-dried, cut into strips of 3mm width and kept at 4°C until use.

### Statistical analysis

The mean values of protein MW were calculated using GraphPad Prism 5.0 (GraphPad Software, California, USA).

## Results

### Analysis and characterization of *T. canis* larvae E/S protein by SDS-PAGE

Our modified culture procedure of *T. canis* L2 larvae in vitro efficiently produced high amount of E/S proteins. After every four weeks of culture, the concentration of E/S protein (Mean ± SD) was approximately 23.9 ± 9.0μg/ml of culture medium (equivalent to 0.85 ± 0.3ng of protein produced per larva per day). The protein components in the E/S products were then determined by SDS-PAGE.

**Fig. 1:**
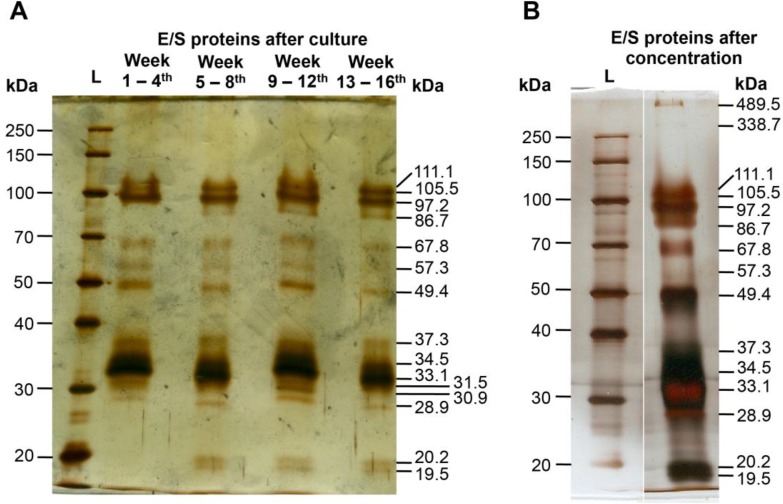
Analysis of E/S product of *T. canis* larvae by SDS-PAGE, 0.2μg of E/S proteins was loaded (A). 2.4μg of E/S proteins was loaded (B). Protein MW was estimated by Quantity One software of at least 3 replicates. L stands for protein ladder.

When 200ng of E/S product was used, we found 15 bands ranging from 19.5 kDa to 111.1 kDa in which the most dominant protein had a MW of 33.1 kDa (Fig. 1A). Several E/S proteins have similar MWs, including four proteins ranging from 30.9 kDa to 34.5 kDa and other two proteins of 105.5 kDa and 111.1 kDa. Unexpectedly, when 2.4μg of E/S product was applied to the gel, we found other two large proteins that have very high estimated MWs of 338.7 kDa and 489.5 kDa (Fig. 1B). There are at least 17 proteins in the E/S products that can be detected by silver staining in SDS-PAGE.

### *T. canis* larvae induce multiple organ inflammations and IgG response in mice

**Fig. 2:**
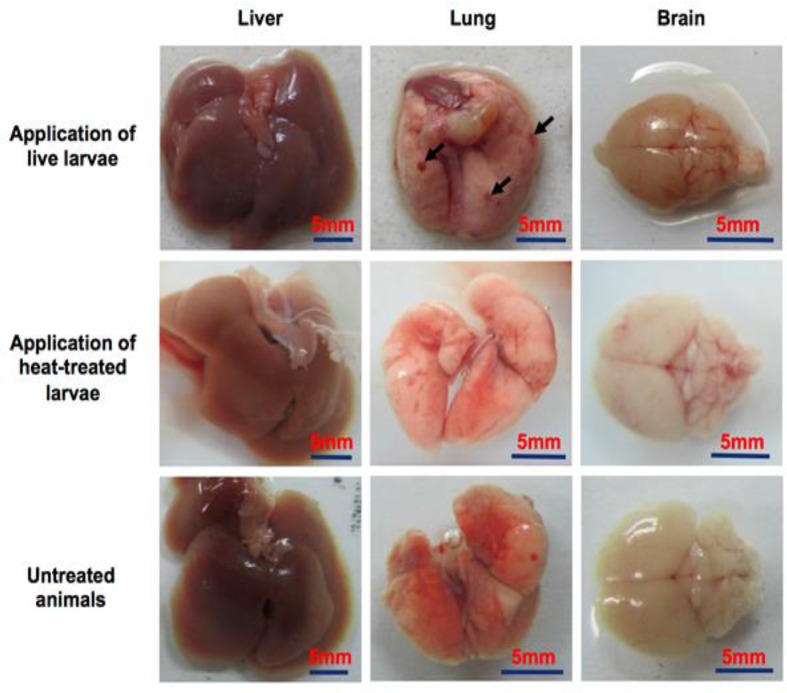
*T. canis* larvae induced lung injury in mice. Arrows show injured spots in indicated tissue. N =5.

**Fig. 3:**
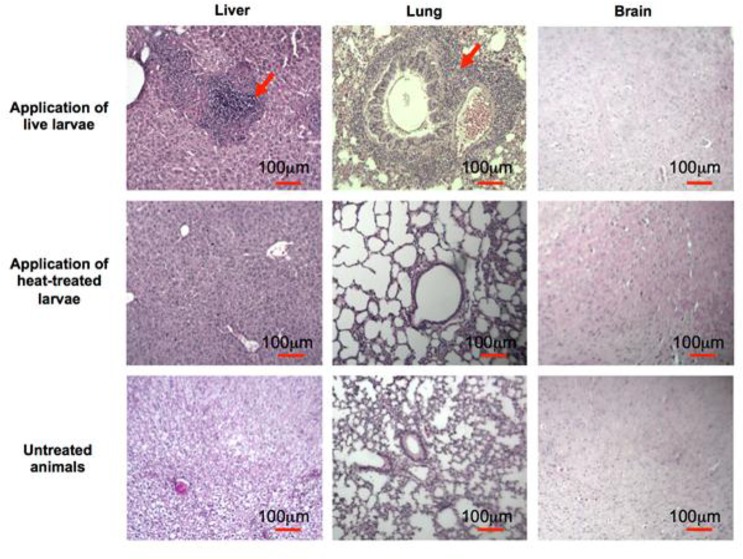
Histological analysis of Giemsa-stained sections of tissues in *T. canis* infected and control mice. Images were obtained at 100X magnification. Arrows show leukocyte accumulation in the indicated tissues. N=5.

After infection, E/S proteins are believed to interact with and manipulate the immune system of the host, though how E/S proteins contribute to this complex interaction is not fully understood. We analyzed the inflammatory response induced by *T. canis* larvae and the humoral immune response of infected mice in order to clarify which E/S proteins result in the generation of an IgG response. Mice were injected ip with 1000 live or dead larvae of *T. canis*. Four weeks later sera and organs (including the liver, the lungs, and the brain) were harvested for histochemical analysis and for the recovery of migrating larvae. Organs from control untreated mice and heat-treated larvae injected mice had no sign of tissue damage, whereas mice treated with live larvae showed an apparent abnormality in the lungs but not in the livers or in the brain (Fig. 2). Histochemistry analysis showed that there is an accumulation of inflammatory leukocytes in both the liver and the lungs but not in the brain in all mice infected with live larvae (Fig. 3). Finally, using the Bearmann procedure we could recover a remarkable number of transferred larvae in the brain (15.6 ± 10.8 larvae per mouse brain, Mean ± SD).

**Fig. 4:**
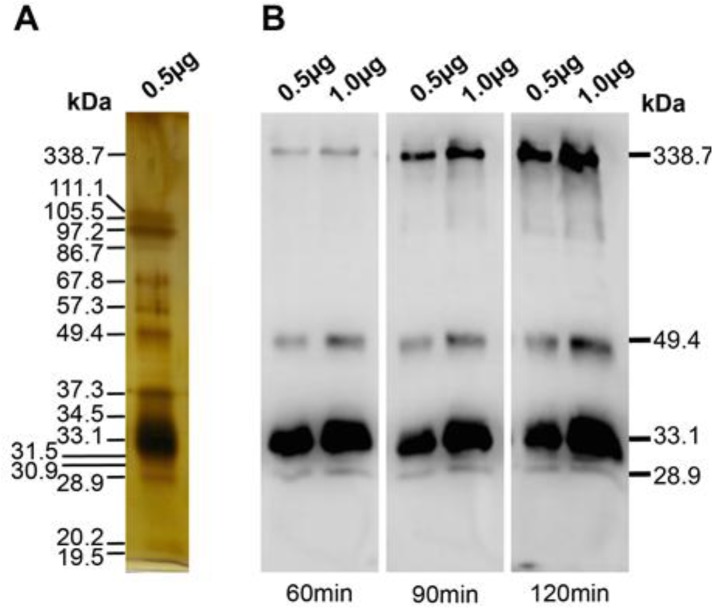
Optimization of blotting time in WB assay, (A) Silver staining of E/S proteins in SDS-PAGE gel. (B) WB band patterns after different blotting times. Data is representative of at least 3 independent experiments.

We next analyzed the IgG response of mice to the E/S product of *T. canis* larvae using the western blotting assay. For optimization, blotting times of 60min, 90min or 120min were used. The 28.9 kDa, 33.1 kDa, 49.4 kDa and 338.7 kDa proteins could be detected by specific IgG in sera of mice infected with live larvae and that 120 min of blotting gave an increasing signal intensity of 338.7 kDa band (Fig. 4).

Unfortunately, we could not clearly detect bands of 97.2 kDa, 105.5 kDa and 111.1 kDa. Staining the gel after blotting showed that these three protein bands of 97.2 kDa, 105.5 kDa, and 111.1 kDa failed to transfer onto the nitrocellulose membrane. They remained in the gel at all blotting times tested (Data not shown). However, the 33.1 kDa protein started to be lost after 120min of blotting (Data not shown) and we, therefore, applied 120min of blotting for all following WB experiments.

**Fig. 5:**
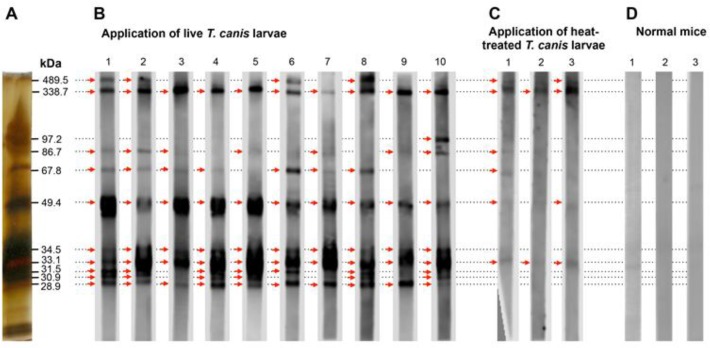
Analysis of IgG response in mice infected with *T. canis* larvae by WB assay. (A) Silver staining of *T. canis* E/S proteins in SDS-PAGE gel before blotting. (B) WB band patterns from sera of mice infected with live *T. canis* larvae. (C) WB band patterns from sera of mice infected with heat-treated *T. canis* larvae. (D), WB band patterns from sera of normal mice. Arrows show positive bands.

To analyze the IgG response in mice, WB strips were prepared from one gel to be sure that each strip contains the same amount of bound antigens. At least 10 bands were detected on the strips using the sera of animals infected with live larvae (N=10) (Fig. 5B). The strongest intensity bands were those at 33.1 kDa, 49.4 kDa, and 338.7 kDa. With the exception of mouse number 10, bands at 97.2 kDa, 105.5 kDa, and 111.1 kDa were not detected and this was probably due to the failure to blot these proteins to the membrane (Fig. 4). In contrast, in mice injected with heat-treated larvae (N = 3), sera only labeled five bands very weakly (Fig. 5C). None of these bands was labeled when tested against the sera of untreated mice (N=3) (Fig. 5D). The heterogeneous WB band patterns could be applied to discriminate mice infected with live larvae from those infected with dead larvae and normal control. The reactivity of each E/S proteins in serodiagnosis of toxocariasis in mice infected with living *T. canis* larvae was determined and the results are shown in Table 1. Five proteins showing the highest reactivity have MWs of 28.9 kDa, 33.1 kDa, 34.5 kDa, 49.4 kDa and 338.7 kDa.

**Table 1:** The reactivity of E/S proteins produced by *T. canis* larvae in serodiagnosis of toxocariasis in mice

**WB bands (kDa)**		**28.9**	**30.9**	**31.5**	**33.1**	**34.5**	**49.4**	**67.8**	**86.7**	**97.2**	**338.7**	**489.5**
Reactivity (%)	*T. canis* infected	100 (10/10)	60 (6/10)	70 (7/10)	100 (10/10)	100 (10/10)	100 (10/10)	70 (7/10)	70 (7/10)	10 (1/10)	100 (10/10)	40 (4/10)
	*T. cati* infected	75 (6/8)	50 (4/8)	50 (4/8)	100 (8/8)	100 (8/8)	100 (8/8)	87.5 (7/8)	87.5 (7/8)	0 (0/8)	87.5 (7/8)	87.5 (7/8)

### E/S product produced by T. canis larvae does not discriminate T. canis and T. cati larvae infection in mice

**Fig. 6:**
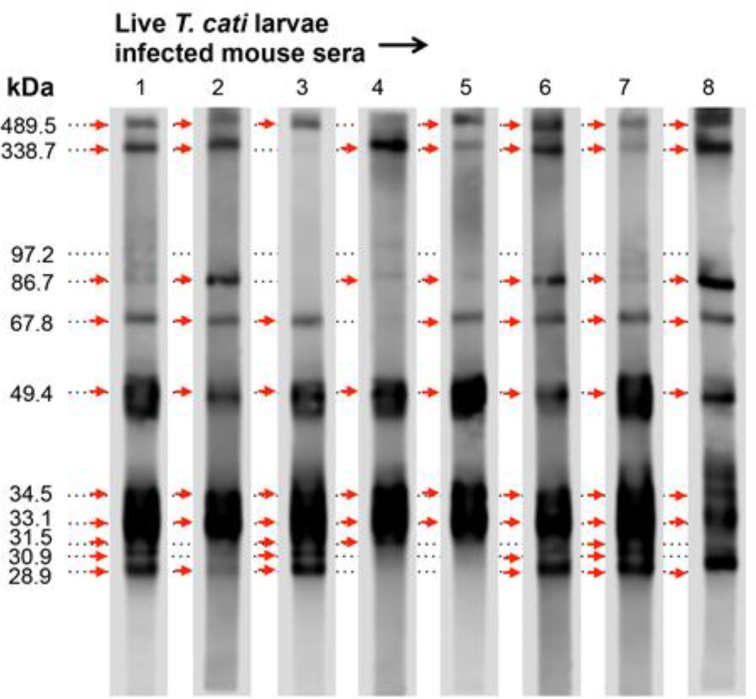
WB analysis of cross-reaction of E/S proteins from *T. canis* larvae with sera from mice infected with live *T. cati* larvae. WB strips were prepared as shown in Fig. 5. Arrows show positive bands.

E/S proteins from *T. canis* larvae strongly cross-react with serum from animals infected with *T. cati* larvae using an ELISA assay (21). Whether each of the E/S proteins plays an equivalent role in this cross-reaction is unclear. To answer the question, a group of eight mice were injected ip with 1000 live *T. cati* larvae. Four weeks later, sera from these mice were collected and analyzed by the WB assay. E/S proteins of *T. canis* larvae strongly cross-react with IgG from the sera of mice infected with *T. cati* (Fig. 6). However, only four proteins (33.1 kDa, 34.5 kDa, 49.4 kDa and 338.7 kDa) showed high reactivity (Table 1). The reactivity of the IgG response to the 28.9 kDa band is much lower in mice infected with *T. cati* than in mice infected with *T. canis*. There is no E/S protein from *T. canis* larvae that can be used to differentiate efficiently between *T. canis* and *T. cati* infection in mice. Because of their high reactivity, the 33.1 kDa, 34.5 kDa, 49.4 kDa and 338.7 kDa E/S proteins derived from *T. canis* larvae are the best potential candidates for serodiagnosis of toxocariasis in mice. However, the differences in the E/S components produced by *T. canis* and *T. cati* larvae, which result in this cross-reaction, are not yet fully understood and requires further investigation.

### IgG response of paratenic rabbits to T. canis and T. cati larvae

**Fig. 7:**
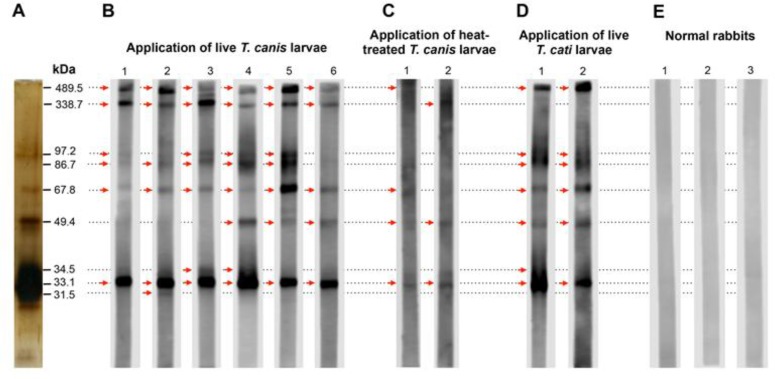
Analysis of IgG response in rabbits 4 weeks after infection with *Toxocara* spp. larvae by WB assay, (A) Silver staining of *T. canis* E/S proteins in SDS-PAGE gel before blotting, (B) WB band patterns from sera of animals infected with live *T. canis* larvae, (C) WB band patterns from sera of animals infected with heat-treated *T. canis* larvae, (D) Cross-reaction of E/S proteins from *T. canis* larvae with IgG in rabbits infected with live *T. cati* larvae, (E) WB band patterns from sera of normal animals, Arrows show positive bands.

We next investigated how rabbits respond to *T. canis* larvae infection and whether the WB band patterns identified in mice are representative for other paratenic hosts. To address this issue, two or six rabbits were respectively infected ip with 1000 heat-treated or live *T. canis* larvae. Four weeks after infection, the IgG response of infected rabbits to E/S proteins was analyzed by the WB assay. Sera from rabbits infected with live larvae bound to the WB with band patterns that mimic those found in infected mice (Fig. 7). We found fewer bands on the WB stained by sera from infected rabbits than from infected mice. In contrast to the sera from infected mice, those from infected rabbits bound principally to the 33.1 kDa band whereas the 28.9 kDa, 30.9 kDa, 31.5 kDa and 35.5 kDa bands were rarely detected. The reactivity of each E/S protein in serodiagnosis of *T. canis* infected rabbits is shown in Table 2. Bands of 33.1 kDa, 67.8 kDa, 338.7 kDa and 489.5 kDa gave 100% reactivity in rabbits while the 49.4 kDa band, which has 100% reactivity in mice, drops to 50% reactivity in rabbits. Unexpectedly, 97.2 kDa protein that had poorly reactivity to infected mouse sera but was readily detected by sera from infected rabbits. Sera from rabbits given dead *T. canis* larvae, formed not only less bands but also very weak bands in comparison to those from animals infected with live larvae (Fig. 7C). Sera collected from normal rabbits (N=3) gave no band in the WB assay (Fig. 7E). We also obtained similar data in two rabbits infected with live *T. cati* larvae though here there was no response to the 338.7 kDa band (Fig. 7D) indicating that the 338.7 kDa protein may be not suitable for serodiagnosis of *Toxocara spp.* infection in rabbits.

**Table 2:** The reactivity of E/S proteins produced by *T. canis* larvae in serodiagnosis of toxocariasis in rabbits

**WB bands (kDa)**		**31.5**	**33.1**	**34.5**	**49.4**	**67.8**	**86.7**	**97.2**	**338.7**	**489.5**
**Reactivity (%)**	T. canis infected	16.7 (1/6)	100 (6/6)	33.3 (2/6)	50 (3/6)	100 (6/6)	83.3 (5/6)	66.7 (4/6)	100 (6/6)	100 (6/6)
	*T. cati* infected	0 (0/2)	100 (2/2)	50 (1/2)	100 (2/2)	100 (2/2)	100 (2/2)	100 (2/2)	0 (0/2)	100 (2/2)

**Fig. 8:**
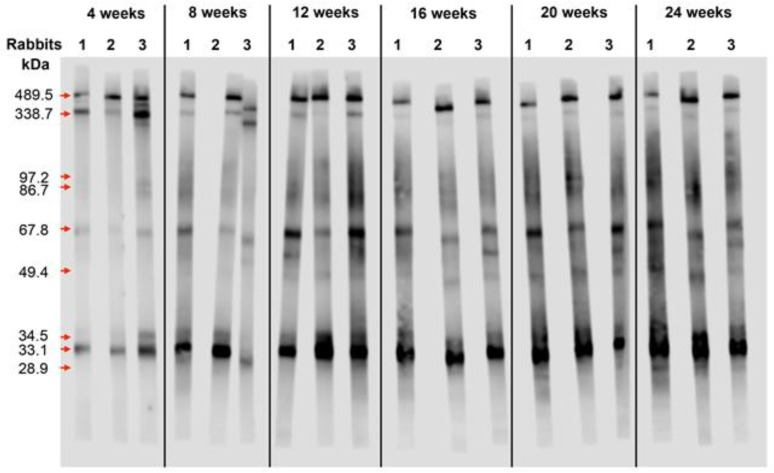
Analysis of IgG response in rabbits during 24 weeks after infection with *Toxocara* canis larvae by WB assay

We next asked whether the WB band patterns works in a time-dependent manner following infection, rabbit sera were collected during 24 wk of infection and IgG response was analyzed. During the course of infection, the WB band patterns are likely unchanged and that the band intensity apparently increases (Fig. 8). The WB band patterns may be time-independent.

Rabbits and mice respond differently to *Toxocara* spp. infection. However, they do share IgG humoral responses to some E/S proteins. The 33.1 kDa E/S protein component induced strong IgG response gave the strongest signal intensity and the highest reactivity in the WB assay for serodiagnosis of toxocariasis in both mice and rabbits.

### IgG response of toxocariasis patients

**Fig. 9:**
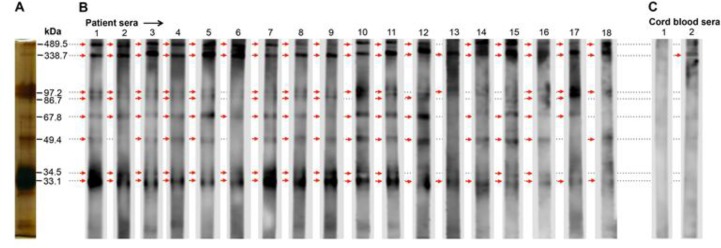
Analysis of IgG response in toxocariasis patients by WB assay, (A) Silver staining of *T. canis* E/S proteins in SDS-PAGE gel before blotting, (B) WB band patterns from toxocariasis patient sera. (C) WB band patterns from cord blood sera. Arrows show positive bands.

Diagnosis of toxocariasis in patients is difficult because of the lack of gold standards. We applied our WB assay to analyze serum from 18 toxocariasis patients whose sera had all typed positive in an ELISA test that used crude adult *T. canis* antigen for coating the plate. For control, we used cord blood sera (N=2). WB band patterns from toxocariasis patients were more similar to those obtained from *Toxocara* spp. infected rabbits than those obtained from *Toxocara* spp. infected mice (Fig. 9). 33.1 kDa and 338.7 kDa bands appeared in all patient sera and yielded a reactivity of 100% (Table 3). However, the 338.7 kDa band was also stained by cord blood sera indicating that this band should not be considered in serodiagnosis of toxocariasis in humans. Consistent with previous demonstration in experimental toxocariasis animal models, our data in human showed that the 33.1 kDa E/S protein can play a key role in determining toxocariasis in human.

**Table 3:** The reactivity of E/S proteins produced by *T. canis* larvae in serodiagnosis of toxocariasis in patients

**WB bands (kDa)**	**33.1**	**34.5**	**49.4**	**67.8**	**86.7**	**97.2**	**338.7**	**489.5**
Reactivity (%)	100 (18/18)	72.2 (13/18)	77.8 (14/18)	88.9 (16/18)	66.7 (12/18)	77.8 (14/18)	100 (18/18)	94.4 (17/18)

## Discussion

In this study, we used the E/S proteins produced by *T. canis* larvae to identify WB patterns that are indicative of *Toxocara* spp. infection in distinct paratenic hosts including mice, rabbits, and human. Characterization of the E/S product by SDS-PAGE showed that it included at least 17 proteins, which had MW ranging from 19.5 kDa to 489.5 kDa. The 33.1 kDa protein made up the most dominant constituent in the E/S product. Our analysis is not identical to that shown previously (26). Some bands appeared in our gel but were absent in Page’s study. These differences might be due to experimental conditions or to differences in the geographical isolates of *T. canis* used (2). This will require further investigation.

In order to establish animal models of toxocariasis, animals are usually orally gavaged with fertilized eggs or larvae (22–24). Larvae then rapidly migrate from the gut lumen and penetrate the gut wall to enter various tissues. Larvae are found early in the liver and later in the lungs and brain (22, 23). In other studies larvae were also injected into the peritoneal cavity and it was shown that the migration behavior of *T. canis* larvae is independent of the route of inoculation (27). In this study, we infected mice by injection of the larvae into the peritoneal cavity. Thirty days after infection, the larvae were recovered from the brain and they had induced leukocyte accumulation and inflammation in the liver and the lungs properly due to their migration to these sites. Intraperitoneal infection allows for normal migration and inflammatory induction and hence that this application route can be used as an effective model of toxocariasis infection. The ip application is rapid and causes minimal stress to the animals.

In our toxocariasis mouse model in which the *T. canis* larvae were injected ip, the E/S proteins induced IgG humoral immune response to different extents in the animals resulting in heterogeneity of the band pattern in the WB assay. Five proteins that have MWs of 28.9 kDa, 33.1 kDa, 34.5 kDa, 49.4 kDa and 338.7 kDa gave the greatest reactivity. The close taxonomic relationship between *T. canis* and *T. cati* results in a high degree of cross-reaction between antibodies raised against E/S proteins from *T. canis* and antibodies from animals infected with *T. cati* (21), but it is not clear whether all E/S proteins contribute equally to the cross-reactivity. The WB patterns detected by sera from *T. cati* infected mice are similar to those detected by sera from *T. canis* infected mice though there is a reduction in the reactivity of the 28.9 kDa band. Alternative approaches will be necessary to discriminate *T. canis* and *T. cati* infections.

In contrast to the situation in mice, rabbits do not mount an effective IgG response to the 28.9 kDa and 30.9 kDa E/S proteins. In addition, the reactivity of the 31.5 kDa, 34.5 kDa and 49.4 kDa bands but not of the 67.8 kDa band was reduced. Paratenic mice and rabbits respond differently to the E/S products of *Toxocara* spp. Once again, we also could not discriminate between *T. canis* infected and *T. cati* infected rabbits. This finding is consistent with previous work that showed an inability to discriminate between these two species in experimentally infected pigs using a WB assay (28).

We provide evidence in this study that the 33.1 kDa E/S protein is not only the major component in the E/S product produced by *T. canis* larvae but it is also the strongest inducer of IgG production and the most sensitive marker in both infected paratenic mice and rabbits.

To determine whether this band would also be of value in the diagnosis of toxocariasis in humans we tested sera from 18 patients all of whom had reacted positively to *T. canis* crude antigen in an ELISA assay. All sera gave WB patterns of *Toxocara* spp. infection similar to those seen in mice and rabbits. Two bands of 33.1 kDa and 338.7 kDa gave 100% reactivity. However, the band at 338.7 kDa was also positive when control cord blood serum was used which indicates low specificity of this signal. In addition, previous work gave evidence that sera from patients infected with a range of other parasites cross-reacted with high molecular weight E/S proteins, whereas low molecular weight E/S proteins (including the 33.1 kDa constituent in our study) show >96% specificity (12). This supports our study for the use of 33.1 kDa E/S protein as the most promising candidate for toxocariasis diagnosis in human.

The 33.1 kDa E/S protein determined in our study is largely equivalent to 32 kDa TES-32 or C-type lectin 1 protein (CTL-1) defined in original studies (16, 26, 29). The shift in its MW is depended on acrylamide concentration of the gel used in electrophoresis procedure (35 kDa at 8% gel and 32 kDa at 12% and 15% gel, our unpublished data). Identification of this protein is in progress. Recombinant CTL-1 and other C-type lectin CTL-2 (which shares 83% amino acid sequence to CTL-1) are able to distinguish *T. canis* infection from other helminth infection in animal model (30). If this is the case, our data then indicate the important role of 33.1 kDa protein (TES-32 or CTL-1) for future applications in immune diagnosis of *Toxocara* spp. infection in both human and animals.

## Conclusion

*T. canis* 33.1 kDa E/S protein is the most reactive constituent recognized by specific IgG in animals and human infected with *T. canis* and/or *T. cati* indicating that this protein may be considered as a critical common biomarker for future applications in diagnosis both veterinary and human medicine. However, further investigation is required to prove its specificity.
